# Evaluating feedback reports to support documentation of veterans’ care preferences in home based primary care

**DOI:** 10.1186/s12877-024-04999-y

**Published:** 2024-05-01

**Authors:** Cari Levy, Jennifer Kononowech, Mary Ersek, Ciaran S. Phibbs, Winifred Scott, Anne Sales

**Affiliations:** 1 Denver-Seattle VA Center of Innovation for Value Driven & Veteran-Centric Care, Rocky Mountain Regional VA Medical Center at VA Eastern Colorado Health Care System, Aurora, CO USA; 2 Division of Geriatric Medicine, University of Colorado Anschutz Medical Campus, Aurora, CO USA; 3 Center for Clinical Management Research, VA Ann Arbor Healthcare System, Ann Arbor, MI USA; 4 Center for Health Equity and Promotion, Corporal Michael J. Crescenz VA Medical Center, Philadelphia, PA USA; 5 Schools of Nursing and Medicine, University of Pennsylvania, Philadelphia, PA USA; 6 Geriatrics and Extended Care Data and Analysis Center, VA Palo Alto Health Care System, Palo Alto, CA USA; 7 Departments of Pediatrics and Health Policy, Stanford University School of Medicine, Stanford, CA USA; 8 Sinclair School of Nursing, Department of Family and Community Medicine, University of Missouri, Columbia, MO USA

**Keywords:** Veteran, Department of Veterans affairs, Advance care planning, Nursing homes, Interrupted time series analysis, Implementation science

## Abstract

**Background:**

To evaluate the effectiveness of delivering feedback reports to increase completion of LST notes among VA Home Based Primary Care (HBPC) teams. The Life Sustaining Treatment Decisions Initiative (LSTDI) was implemented throughout the Veterans Health Administration (VHA) in the United States in 2017 to ensure that seriously ill Veterans have care goals and LST decisions elicited and documented.

**Methods:**

We distributed monthly feedback reports summarizing LST template completion rates to 13 HBPC intervention sites between October 2018 and February 2020 as the sole implementation strategy. We used principal component analyses to match intervention to 26 comparison sites and used interrupted time series/segmented regression analyses to evaluate the differences in LST template completion rates between intervention and comparison sites. Data were extracted from national databases for VA HBPC in addition to interviews and surveys in a mixed methods process evaluation.

**Results:**

LST template completion rose from 6.3 to 41.9% across both intervention and comparison HBPC teams between March 1, 2018, and February 26, 2020. There were no statistically significant differences for intervention sites that received feedback reports.

**Conclusions:**

Feedback reports did not increase documentation of LST preferences for Veterans at intervention compared with comparison sites. Observed increases in completion rates across intervention and comparison sites can likely be attributed to implementation strategies used nationally as part of the national roll-out of the LSTDI. Our results suggest that feedback reports alone were not an effective implementation strategy to augment national implementation strategies in HBPC teams.

## Background

Home Based Primary Care (HBPC) in the Department of Veterans Affairs (VA) provides comprehensive, interdisciplinary care to Veterans with complex chronic disease in their homes [1, 2]. HBPC teams cover wide geographic areas, particularly in the western U.S., and even in more densely populated areas, work quite independently because of the nature of home-based care. Given the severity of illness of Veterans receiving home based care, documenting care preferences for life sustaining treatments (LSTs) such as cardiopulmonary resuscitation, mechanical ventilation, antibiotics, and medically administered nutrition and hydration, is a priority [3]. In 2017, the Veterans Health Administration (VHA) National Center for Ethics in Health Care launched the Life Sustaining Treatment Decisions Initiative (LSTDI), a comprehensive program to elicit goals of care and to identify preferences for LST for Veterans with serious illness [4]. These preferences are documented in a standardized LSTDI note template and durable medical orders that are accessible across the VA health care system. The LST template consists of eight fields, four of which were mandatory at the time this study was conducted to include: decision making capacity; goals of care (e.g., “to be cured” or “to be comfortable”); oral informed consent for the LST plan; and preference for cardiopulmonary resuscitation (“CPR”) status and other LSTs.

*Implementing Goals of Care Conversations with Veterans in VA Long-Term Care Settings* (LTC QUERI) was conducted as a part of the VA Health Services Research and Development (HSR&D) Quality Enhancement Research Initiative (QUERI) program. This QUERI project was designed to support implementation of the LSTDI in long-term care settings, including Home-Based Primary Care and Community Living Centers (CLCs). We specifically focus on our work in HBPC in this paper, and have reported the results of our CLC work previously [5]. 

Audit with feedback involves aggregating clinical or other performance data and providing the data to individual practitioners, teams, or healthcare organizations. It has been studied extensively as an approach to modifying behavior of providers [6–10]. The purpose of this analysis was to compare LST documentation in HBPC programs that received feedback reports to matched programs that did not receive feedback reports. We hypothesized that despite modest benefits documented in the literature related to audit with feedback reports as an implementation strategy, this might be an effective approach given the familiarity HBPC providers have with conducting goals of care conversations (GoCC) and documenting Veterans LST preferences. In addition, we anticipated that the initiative would be highly salient, and LST documentation would be high in this population because Veterans receiving HBPC services are frail and at high risk of life-threatening conditions [11]. 

## Methods

The LSTDI was released in January 2017, and all VA health care facilities were mandated to implement the LSTDI program by July 2018. We created monthly feedback reports depicted in tabular and graphical form that showed the number and percentage of Veterans in the program with a documented LST template. Feedback reports were initially sent to selected champions in intervention sites quarterly in April 2018 and increased in frequency to monthly beginning in October 2018. Feedback reports were sent to HBPC programs through February 26, 2020.

### Sources of participants and data

The 13 HBPC programs that received feedback reports were in three Midwestern states and four Western states, located across two VA regional networks. HBPC programs represented a convenience sample selected to participate due to proximity to LTC QUERI team members. Table 1 includes data showing regional distribution and urban/rural locality.

**Table 1:** Characteristics of HBPC Invention and Comparison Sites

Characteristics	HBPC Sites		
	Intervention (*n* = 13)	Matched (*n* = 26)	Significance
**Continuous variables**	**Mean (Std. dev.)**		
Age in years	78.0 (2.3)	78.3 (1.8)	NS
Race/Ethnicity: White (%)	90.8% (6.3)	87.2% (17.5)	NS
Highest Military Priority Status (%)	26.0% (8.9)	26.7% (5.8)	NS
Deceased during fiscal year (%)	18.2 (4.5)	18.6 (3.1)	NS
JEN Frailty Index Score	5.9 (0.4)	5.6 (0.4)	*p* < 0.05
Number of ADL Dependencies	2.1 (0.7)	2.0 (0.7)	NS
NOSOS Score sing VA HCC Score †	5.0 (0.5)	4.8 (0.6)	NS
Care Assessment Need Score (1-year probability of death or hospitalization)	87.3 (1.4)	86.9 (2.6)	NS
HBPC Full-time Equivalents	15.3 (7.0)	17.5 (8.5)	NS
**Categorical variables**	**Percent**		
Primary Care Provider based at Medical Center rather than embedded in HBPC (%)	15.4	15.4	NS
*Rural-Urban Description*			
1: Urban/Metro Area of > = 250,000	15.4%	26.9%	NS
2: Urban/Metro Area of < 250,000	53.8%	50.0%	NS
3: Rural/Adjacent to Metro Area	15.4%	7.7%	NS
4: Rural/Non-Adjacent to Metro Area	15.4%	15.4%	NS

Footnote: All continuous variables were included in the Principal Component Analysis except where indicated; Urban and rural classifications from USDA Economic Research Service; Geographic Divisions from U.S. Census Bureau

NS – not statistically significant; *p < 0.1, **p < 0.05, † Concurrent model, ‡ Not included in Principal Component Analysis

HCC = Hierarchical Condition Category; ADL = Activities of Daily Living; HBPC = Home Based Primary Care

### Implementation intervention - feedback reports

The feedback reports were developed iteratively using user-centered design methods prior to the LSTDI national roll-out in January 2017 [12]. Five sites participated in the user-centered design process with LTC QUERI team members. Four of the sites served as demonstration sites for the national creation of the LSTDI and one site was selected due to proximity to LTC QUERI team members, which provided an opportunity to conduct in-person meetings. All five sites had an HBPC program. The user-centered design cycle occurred over approximately 18 months and 12 iterations prior to finalizing the feedback reports.

HBPC census data for the implementation sites were identified using the HBPC Master File, a VA national database. Veterans who had an outpatient encounter in an HBPC clinic in the past 30 days were included as part of the census. Completed LST templates were identified as those with all four required questions completed, as these questions need to be completed for the template to translate to an order set in the electronic medical record. We identified GoCCs that took place at any time prior to the HBPC admission date, as well as those that occurred on the first or second HBPC visit.

Feedback reports were sent via email each month to the site champion(s) for each HBPC program in the intervention. VA clinicians and teams often receive direct performance feedback, but we made the decision to utilize site champions based on prior research on audit with feedback that has shown that it is most effective when delivered by a superior or respected colleague [7]. Each site identified one or two HBPC team members to serve as the site champion. Site champions were identified as leaders within their HBPC program and included HBPC program directors, nurses, social workers, and providers. Site champions were reminded in the feedback report email and during qualitative interviews that the feedback reports could be shared with their HBPC teams and leadership, and the site champions ultimately had control of whether the reports were distributed widely to staff, leadership or not shared beyond the champion(s).

The feedback reports showed the number of LST templates completed before admission to HBPC, at 1st HBPC visit, 2nd visit, or 3rd visit or later (Appendix 1). These time points were selected based on user input and the realities of home-based care; clients are often not seen on a regularly scheduled basis, so time-based metrics such as within 14 days were not felt to be appropriate.

### Matching

We matched intervention to comparison HBPC programs using Euclidean distance calculated between scores for each HBPC program derived by a principal component analysis (PCA), a factor analytic approach reducing a large number of variables to a small number of scores from retrospective data [13, 14]. The identified comparison HBPC sites were also discussed with the National HBPC Program Manager to ensure there were no additional factors that would preclude them from being an appropriate match. Details of the matching process and the use of PCA to create a score are provided in a previous paper [5]. 

The thirteen intervention HBPCs were matched with twenty-six non-intervention HBPCs. Aggregated HPBC level variables capturing site and patient population characteristics in fiscal year 2018 were used: age on October 1, 2018; average number of ADL dependencies; Care Assessment Need score (1-year probability of hospitalization or death) [15, 16]; number of full-time employees in the HBPC program; JEN Frailty Index score [17]; estimated cost (a NOSOS score of 1 means the Veteran is expected to have costs that are the national average for VA patients) [18]; and the proportions of patients who were married; died during the fiscal year, and/or were identified as white. These variables were used in a principal components analysis resulting in four principal component scores. The scores and the number of admitted patients were used to calculate the Euclidean distances between the intervention and non-intervention HBPCs. The intervention HBPCs were matched with the two closest non-intervention sites and no non-intervention site was matched with more than one intervention site. Sites were also matched based on urban/rural classification and HBPC primary care provider (PCPs) structure with either HBPC-embedded PCPs or medical center PCPs. These variables were compared between the intervention and matched sites using T-Tests and Wilcoxon rank-sum tests to confirm that there were no significant differences between the groups.

### Outcome measures

The primary outcome was the proportion of Veterans with completed LST templates aggregated to the level of the HBPC program at either the first or second HBPC visit or prior to admission. Specific data elements called LST health factors were retrieved from the VA Corporate Data Warehouse (CDW) in order to identify who had a documented LST template. These data were merged with data on Veterans in HBPC, and then aggregated on a bi-weekly basis over the period between March 1, 2018, and February 26, 2020, for all intervention and separately for all matched sites.

### Statistical analysis

Interrupted time series analysis was used to estimate the effect of the feedback reports on the LST completion rates. The interruption point was set at the week of July 5, 2018, which was the point at which all sites were expected to have fully implemented the initiative [4]. This gave us nine time points before the implementation time point, and 43 time points after. Coefficients were estimated for the variables included in the interrupted time series model (intercept, trend before interruption, change in level, and trend after interruption) and represented in time series graphs.

Analyses were conducted using SAS statistical software, version EG 7.1 to match the sites using PCA. R Studio, running R version 4.0.2, was used for the interrupted time series analysis with statistical significance at *p* < 0.05.

### Process evaluation

We conducted episodic interviews with the sixteen site champions throughout the intervention period to assess barriers and facilitators to implementing the LSTDI, as well as gauge the use and distribution of the feedback reports. The interview data was analyzed using the Consolidated Framework for Implementation Research (CFIR) [19]. To determine the reach of the feedback reports we also deployed a post-feedback survey using REDCap, a web-based survey platform. We distributed the survey on a quarterly basis to HBPC team members including the site champions. The site champions and HBPC leadership provided the LTC QUERI team with a list of staff email addresses to invite to complete the survey. Between 12 and 60 HBPC team members received the email invitation to complete the survey each quarter. The five questions related to the feedback report, and included whether the respondent: received the report, read it, understood it, found it useful, and if they discussed it with other staff. Descriptive statistics (means and standard deviations) describe the data. As a secondary process outcome measure, we also compared the proportion of LST templates completed prior to HBPC enrollment, in other VA care settings, such as hospital admission or primary care, to identify the proportion of LST templates that were completed after admission to HBPC.

Our study was deemed quality improvement (QI) by the VA Research and Development Committee, exempt from human subject’s oversight, and exempt from obtaining informed consent.

## Results

### Matching

Veteran and HBPC program characteristics for intervention (*N* = 13) and comparison sites (*N* = 26) are presented in Table 1. The mean Euclidian distance between intervention and matched sites was 1.24 with a standard deviation of 0.73. The results are reported for both continuous and categorical variables in Table 1. There were no statistically significant differences between the intervention and comparison sites in terms of mean Veteran age in years (78.0 ± 2.3 vs. 78.3 ± 1.8, respectively), race/ethnicity (90.8% ± 6.3% White for intervention sites vs. 87.2% ± 17.5% White for comparison sites), or highest military priority status (26.0% ± 8.9% for intervention sites vs. 26.7% ± 5.8% for comparison sites). Mortality rates during the fiscal year did not differ significantly (18.2% ± 4.5% for intervention sites vs. 18.6% ± 3.1% for comparison sites), nor did the number of ADL dependencies (2.1 ± 0.7 for intervention sites vs. 2.0 ± 0.7 for comparison sites), VA Hierarchical Condition Category (HCC) scores (5.0 ± 0.5 for intervention sites vs. 4.8 ± 0.6 for comparison sites) or care assessment need scores (87.3 ± 1.4 for intervention sites vs. 86.9 ± 2.6 for comparison sites). HBPC full-time equivalents were slightly higher in the comparison sites (15.3 ± 7.0 for intervention sites vs. 17.5 ± 8.5 for comparison sites; NS) and a statistically significant difference was found in the JEN Frailty Index Score, with the intervention sites having a higher score compared to the comparison sites (5.9 ± 0.4 vs. 5.6 ± 0.4, *p* < 0.05). The rural-urban description showed similar distribution patterns between the two groups, with no significant differences observed across the four categories.

Table 2 presents the interrupted time series analysis results. The baseline level of completed LST templates was estimated to be 4.26%, with a standard error of 2.48, not statistically significant. The coefficient on the pre-interruption trend had a coefficient estimate of 1.01, with a standard error of 0.52 (*p* = 0.06). The change in level at the beginning of the feedback intervention was estimated to be 3.99%, with a standard error of 3.18, not statistically significant, indicating no significant change in the level at the initiation of the feedback intervention. Similarly, the change in the trend after the interruption showed a coefficient estimate of -0.65, with a standard error of 0.52 which did not reach statistical significance, suggesting that there was no significant change in the trend after the intervention. The dummy variable comparing between the intervention and comparison groups had a coefficient estimate of -0.88, with a standard error of 3.51 with no significant difference between the two groups. Furthermore, the differences between the groups in the prior trend, change in level, and change in trend were not statistically significant. In sum, there were no statistically significant effects of the intervention at the point of interruption in the segmented regression analysis.

**Table 2:** Interrupted Time Series Results

	Coefficient estimate	Standard error	Significance *p*-value
Baseline level of completed LST template	4.26	2.48	0.0896
Trend before interruption	1.01	0.52	0.0570
Change in level from beginning of feedback intervention	3.99	3.18	0.2119
Change in trend after feedback intervention began	-0.65	0.52	0.2198
Dummy variable for group: intervention vs. matched comparison	-0.88	3.51	0.8019
Difference between groups in prior trend	-0.02	0.74	0.9788
Difference between groups in change in level	-4.63	4.49	0.3046
Difference between groups in change in trend	0.28	0.74	0.7024

* *p* < 0.01, ** *p* < 0.05

**Fig. 1:**
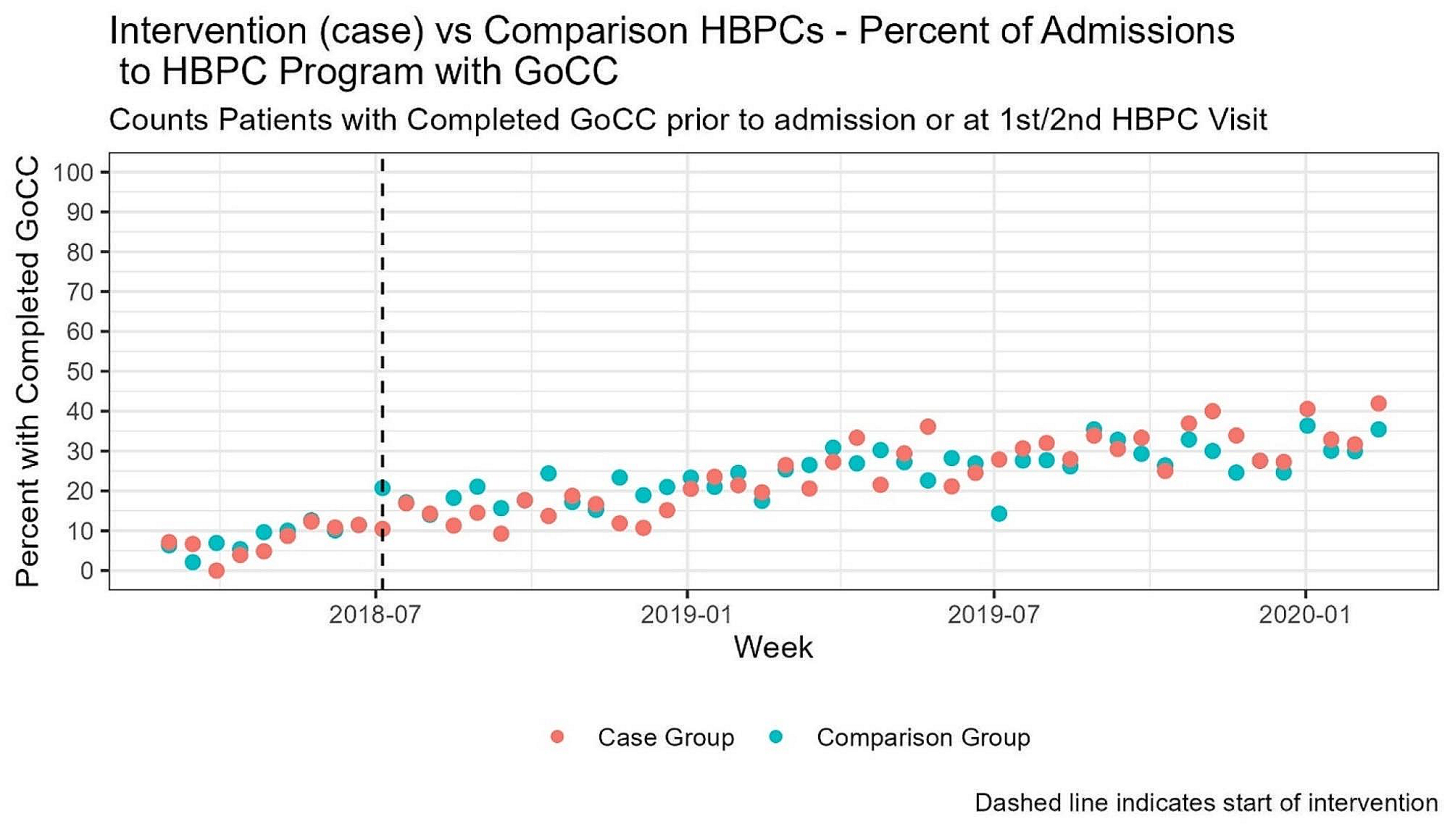
Intervention vs. Comparison Data: Percent of HBPC Admissions with Life-Sustaining Treatment Documentation Completed During the 1st or 2nd HBPC Visit or Prior to Admission by Bi-Weekly Interval

Figure 1 presents the comparison between the intervention and comparison groups regarding the percent of HBPC admissions with completed life-sustaining treatment documentation. The data are displayed over bi-weekly intervals to provide a visual representation of the differences between the two groups over time. The figure illustrates that the trends in the completion of life-sustaining treatment documentation during the 1st or 2nd HBPC visit or prior to admission were low for both intervention and matched comparison sites prior to the intervention 6.34% and 7.14% respectively. While these rates increased in the intervention sites to 35.4%, comparison sites increased to 41.9%, no significant differences were noted (Fig. 1).

### Process evaluation results

Feedback report surveys to team members indicated that feedback reports were not distributed widely to HBPC team members or leadership. The survey was completed a total of 174 times across the four quarters, with a response rate between 9.4% and 19.3% [20]. The feedback report survey found that between 12.8% and 25.5% of HBPC team members who completed the survey reported receiving the feedback report from their site champion.

Through our episodic semi-structured interviews and email correspondence with site champions (N = ∼ 50) we heard multiple reasons why the feedback reports were not distributed widely. These reasons included the belief that the reports showed discouraging results for newly enrolled Veterans.

"Like I said, we have people in our program who have been with our program for 5 years, and you’d never capture that we had the conversation with them by only pulling new admissions."

"I know you’re not using the data in any punitive way, but when I see one documented and I know I’ve had several, I feel a little defensive."

They also reported concerns that the reports only showed rates of completed LST note and order templates which required signature by prescribing providers and did not reflect documentation of GoCCs completed by other disciplines such as social work, nursing, and chaplaincy.

"Prescribing providers are not documenting LST templates in our HBPC program, this is being done by Social Workers. So, the reports show that no LST templates are being completed, but they are being completed by non-prescribing providers."

Finally, champions indicated that they felt the feedback reports would discourage staff as they showed low LST template completion rates.

"I wouldn’t show this report today because it looks like we’re not doing anything, and I don’t want anyone to think that someone is tracking that we’re not doing the right thing."

## Discussion

The aim of this study was to investigate whether the use of monthly feedback reports resulted in improvements in LST template completion for HBPC residents. Our findings revealed there were no significant positive differences between intervention and comparison sites solely from the use of feedback reports. Increases in LST template completion rates for both intervention and comparison HBPC teams between March 2018 and February 2020 are likely due to the well-organized national roll-out of the LSTDI by the National Center for Ethics in Health Care, as well as expected increases that occur after the release of an initiative [11]. Our process evaluation uncovered potential reasons for the failure of feedback reports to significantly increase LST template completion, including concerns shared by site champions that they worried sharing the reports would discourage staff because they felt the rate of LST template completion was low. Site champions also expressed concern that the feedback reports did not provide a complete picture of the effort to conduct goals of care conversations with inclusion of data only from prescribing providers and not for the non-prescribing provider template that is often used to inform LST template completion [21, 22]. While stakeholders from HBPC programs were included in the design of the feedback reports, these concerns were not identified during the design process suggesting that iterative adaptation may be needed throughout the implementation period rather than isolating refinement to the pre-implementation period [6]. 

In a previously published paper, we found similar results for the effects of a feedback intervention only compared to a more extensive intervention that coupled feedback reports with coaching and other activities in VA Community Living Centers (CLCs), which are VA-owned and operated long term care facilities [5]. Our core finding was that there was an effect with a more extensive intervention, whereas the feedback intervention alone did not result in improved outcomes. However, in that work, we found much higher rates of documentation of goals of care conversations through the LST template. There are likely many explanations for this, but key differences between institutionalized care, where Veterans are congregated and can receive daily attention and care from health care providers, and home-based care, in geographically dispersed and diverse settings, are an important factor. HBPC teams may not see each other more than once a week, and coordination among care providers is complex. In residential, institutional settings, coordination is less complex.

Based on our findings, we recommend coupling additional facilitation activities with audit and feedback reports to impact behavior change such as peer comparisons, education or coaching [23]. Facilitators can offer expert guidance based on clinical experience, address barriers based on their real-world experience, and provide tailored strategies to overcome challenges specific to the healthcare setting of interest [8]. Peer facilitators can create a collaborative learning environment, promote engagement by staff and model a culture of continuous quality improvement. While feedback reports serve as the foundation for reflection on the data, facilitation provides guidance to translate those insights into an action-oriented approach [24]. 

These findings informed design by the LTC QUERI team of a program entitled Preferences Elicited and Respected for Seriously Ill Veterans (PERSIVED) to extend their work in HBPC and community nursing homes [25]. In this ongoing project, implementation facilitation is utilized to address problems and offer customized support adapted to the context and individual characteristics of the recipients of the intervention [26, 27]. PERSIVED sites receive monthly feedback reports that show their rates of LST template completion, as well as monthly coaching sessions with PERSIVED facilitators. The coaching sessions are led by a former HBPC program director and a social worker who have extensive knowledge of the context of HBPC programs and conducting GoCCs and documenting LST templates. By adding the additional support of implementation facilitation our hope is that audit with feedback will be more widely used and distributed which will lead to an increase in LST template completion.

There were several limitations to this study. First, our study was conducted with a small number of HBPC programs chosen based on proximity to LTC QUERI team members as a convenience sample. This may have resulted in a biased sample and limits the generalizability of the findings based on the limited geographic variability. Second, the site champions decided whether to distribute the feedback reports and, if distributed, to whom. Based on our survey findings and qualitative interviews with site champions, we found that the feedback reports were not widely distributed. Now knowing that the champions shielded reports, we should have asked pointed questions during the user-centered design cycle about what champions would do if their feedback report data was not “favorable” and worked with champions to overcome this barrier prior to deploying the feedback reports. Third, due to a delay in the release of the LSTDI policy, there were fewer time points to measure and a shorter intervention period for programs to adapt. Additionally, the COVID-19 pandemic may have impacted the implementation and effectiveness of the intervention towards the end of the project due to competing demands for clinician time and shifting priorities. Finally, we note that there were limited sites available to use as matching sites.

## Conclusions

In summary, the results of this feedback intervention in HBPC to ensure that seriously ill Veterans have care goals and LST decisions elicited and documented indicate that the feedback intervention did not have had a significant impact on the completion of LST documentation as measured in this study. Observed increases in documentation occurred in both intervention and comparison programs which is likely attributable to the national implementation efforts with no observed augmentation with feedback reports. Feedback reports may need augmentation with other implementation strategies to change practice behavior.

## Data Availability

The datasets used and/or analyzed during the current study are available from the corresponding author on reasonable request.

## Abbreviations

CDW: Corporate Data Warehouse
CFIR: Consolidated Framework for Implementation Research
CLC: Community Living Centers
CPR: Cardiopulmonary resuscitation
GoCC: Goals of Care conversations
HBPC: Home Based Primary Care
HCC: VA Hierarchical Condition Category
HSR&D: VA Health Services Research and Development
LSTDI: Life Sustaining Treatment Decisions Initiative
LST: Life Sustaining Treatments
LTC: Long Term Care
PCA: Principal component analysis
PCPs: Primary Care Providers
PERSIVED: Preferences Elicited and Respected for Seriously Ill Veterans
QI: Quality improvement
QUERI: Quality Enhancement Research Initiative
VA: Department of Veterans Affairs
VHA: Veterans Health Administration
